# Biosensors in Microbial Ecology: Revolutionizing Food Safety and Quality

**DOI:** 10.3390/microorganisms13071706

**Published:** 2025-07-21

**Authors:** Gajanan A. Bodkhe, Vishal Kumar, Xingjie Li, Shichun Pei, Long Ma, Myunghee Kim

**Affiliations:** 1 Department of Food Science and Technology, Yeungnam University, Gyeongsan-si 38541, Gyeongsangbuk-do, Republic of Korea; gabodkhe@yu.ac.kr (G.A.B.); vishalkumar@yu.ac.kr (V.K.); 2 State Key Laboratory of Food Nutrition and Safety, Key Laboratory of Industrial Microbiology, Ministry of Education, College of Biotechnology, Tianjin University of Science & Technology, Tianjin 300457, China; 13943484402@163.com (X.L.); malong@tust.edu.cn (L.M.); 3 Jilin Shuangzheng Diagnostic Monoclonal Antibody Scientist Studio, Surge Medical Inc., Tonghua 134000, China; peishichun@thnu.edu.cn; 4 Antibody Development Jilin Province University Enterprise Joint Technology Innovation Labs, Tonghua Normal University, Tonghua 134000, China

**Keywords:** microorganisms, biosensors, food safety, food quality

## Abstract

Microorganisms play a crucial role in food processes, safety, and quality through their dynamic interactions with other organisms. In recent years, biosensors have become essential tools for monitoring these processes in the dairy, meat, and fresh produce industries. This review highlights how microbial diversity, starter cultures, and interactions, such as competition and quorum sensing, shape food ecosystems. Diverse biosensor platforms, including electrochemical, optical, piezoelectric, thermal, field-effect transistor-based, and lateral flow assays, offer distinct advantages tailored to specific food matrices and microbial targets, enabling rapid and sensitive detection. Biosensors have been developed for detecting pathogens in real-time monitoring of fermentation and tracking spoilage. Control strategies, including bacteriocins, probiotics, and biofilm management, support food safety, while decontamination methods provide an additional layer of protection. The integration of new techniques, such as nanotechnology, CRISPR, and artificial intelligence, into Internet of Things systems is enhancing precision, particularly in addressing regional food safety challenges. However, their adoption is still hindered by complex food matrices, high costs, and the growing challenge of antimicrobial resistance. Looking ahead, intelligent systems and wearable sensors may help overcome these barriers. Although gaps in standardization and accessibility remain, biosensors are well-positioned to revolutionize food microbiology, linking ecological insights to practical solutions and paving the way for safer, high-quality food worldwide.

## 1. Introduction

From a public health perspective, food safety, shelf life, and sensory quality are profoundly influenced by microbial ecology, whereby the dynamic interplay of diverse microbial communities is recognized as a cornerstone of food systems [1,2]. Beneficial microbes, such as *Lactococcus* spp. and *Lactobacillus* spp., drive fermentation in dairy and meat products, enhancing flavour and achieving natural preservation [1,2]. Conversely, pathogens like *Listeria* spp. and *Escherichia coli* pose significant health risks if undetected [3,4]. Traditional detection methods, including culture-based techniques, polymerase chain reaction (PCR), and microscopy, are effective but time-intensive, often requiring days to yield results, amplifying risks in modern food production [5,6]. Biosensors address these shortcomings by integrating biological recognition elements, such as antibodies or aptamers, with advanced transducers for real-time microbial detection. These deliver speed and precision, unattainable by conventional approaches, detecting *E. coli* O157:H7 in 20 min using a microelectrode array [7] and identifying *Salmonella* spp. through nucleic acid-based sensors [8]. Calorimetric detection of *Lactobacillus plantarum* was achieved in 4.7–18.6 h [9]. Advancements like nanomaterials and IoT connectivity envision intelligent, responsive food safety systems [10]. However, challenges such as high costs and complex food matrix interference persist [11], suggesting broader adoption depends on overcoming these barriers.

This review primarily focuses on the critical role of biosensors in food microbiology, in which microbial ecology and technological innovation have been seamlessly combined to enhance global food safety from a public health perspective. Foundational studies emphasise the importance of biosensors in analyzing complex and dynamic microbial communities, positioning them as essential tools for monitoring food system microbiomes. Applications—from rapid pathogen detection in produce to real-time spoilage tracking in meat via volatile compounds—are highlighted [7,8]. Limitations such as cost and specificity were evaluated, yet bold future pathways, including wearable sensors and antimicrobial resistance (AMR) monitoring, are envisioned as transformative frontiers. Through this lens, biosensors are cast as the vital link whereby ecological insights are translated into actionable safeguards, thereby elevating food quality and public health worldwide.

## 2. Starter Cultures

Microorganisms selected as starter cultures were deliberately introduced into food matrices to guide fermentation, ensuring consistent quality, improving sensory attributes, and enhancing food safety [Leroy and De Vuyst]. LAB (e.g., *Lactococcus* and *Lactobacillus)* and *Streptococcus* spp. dominated dairy fermentations, while yeasts such as *Saccharomyces cerevisiae* drive the production of bread and beverages [12]. These cultures excelled when they rapidly acidified the matrix, tolerated diverse processing conditions, and maintained stability across repeated cycles, as emphasised by Liu et al. (2023) [13]. Compatibility among co-cultured strains proved essential, as it prevented antagonism and allowed for harmonious fermentation [14]. Some cultures, such as certain Lactobacillus spp., serve as probiotics, conferring health benefits, although safety, efficacy, and stability are critical [15,16]. Biosensors monitor these cultures in real-time, tracking L. plantarum activity in 4.7–18.6 hours via isothermal microcalorimetry, ensuring fermentation success and probiotic viability [17].

## 3. Composition of Food Microbial Communities

Food systems host diverse microbial ecosystems that influence food safety and quality. These microbiomes are shaped by numerous factors, including food type, processing, and environmental factors [3]. This section examines microbial diversity across various food categories, the ecological forces that drive these communities, and the tools used to analyse them, highlighting biosensors as crucial for real-time profiling. These devices delivered rapid insights into microbial composition and activity, supporting proactive safety measures and deepening ecological understanding.

### 3.1. Microbial Diversity Across Food Types

Microbial communities vary widely across food systems, their unique ecological niches dictating safety and quality outcomes. In dairy, lactic acid bacteria (LAB), such as Lactobacillus and Streptococcus, drive fermentation, coexisting with spoilage yeasts and pathogens, including *Listeria* [2,18]. Meat products harbour LAB alongside spoilage agents, such as Pseudomonas, and pathogens, including Salmonella and Campylobacter, with storage conditions modulating their prevalence [3,19]. Fresh produce supports the growth of *E. coli*, *Salmonella* spp., and fungi, posing risks through soil or water contamination [20]. Biosensors swiftly detect this diversity, with surface plasmon resonance (SPR) sensors detecting *Salmonella* spp. in real time [21] and microelectrode arrays detecting *E. coli* O157:H7 in 20 min [7].

### 3.2. Factors Shaping Microbial Ecology

A complex interplay of intrinsic and extrinsic factors governed microbial ecology in food, defining growth and activity [3]. Acidic dairy favours *Lactobacillus* spp., while low water activity in dried foods supports molds [2]. Refrigeration promotes psychrotrophic bacteria, and modified atmosphere packaging shifts the aerobic–anaerobic balance [22]. Electrochemical biosensors track pH changes and volatile compounds, signalling microbial activity for quality control [23].

### 3.3. Tools for Microbial Analysis

Analyzing food microbial communities requires a suite of tools that uncover their diversity and hazards with distinct strengths. Culture-based methods miss viable but nonculturable (VBNC) microbes [17]. PCR detects specific [5], 16S rRNA sequencing profiles of broader communities [6], and metagenomics reveals both composition and function [24]. Biosensors complement these with real-time detection, with aptasensors identifying *Salmonella* spp. [25], Quartz crystal microbalance (QCM) sensors detecting *Staphylococcus* spp. via mass changes [26,27] and nucleic acid-based sensors tracking *Salmonella* spp. [8]. Table 1 summarizes the types of microbes, their roles, and examples of biosensor detection.

## 4. Microbial Interactions and Their Effects

Microbial interactions—encompassing competition, cooperation, and quorum sensing (QS)—have a profound influence on food quality and safety by shaping community dynamics [1]. Biosensors provide critical, real-time insights into these processes, linking ecological complexity to practical outcomes. This section illustrates how these interactions shape food ecosystems and highlights the role of biosensors in monitoring them, offering a window into microbial behavior that enhances food safety and quality control.

### 4.1. Competition

Within food microbial ecosystems, competition naturally suppresses pathogens, as observed with LAB in cheese production. These bacteria produce bacteriocins and lower pH to outcompete *Listeria*, bolstering safety and preservation [2]. Electrochemical biosensors detect these antimicrobial metabolites, thereby confirming pathogen suppression, a capability lacking in traditional methods [29]. This real-time monitoring reveals competitive dynamics, strengthening food safety strategies.

### 4.2. Cooperation

This cooperative interaction mechanism increases fermentation output, which can be seen in the yogurt-making process, where Streptococcus thermophilus and Lactobacillus bulgaricus encourage each other’s growth and metabolism [30]. This synergy ensures a consistent texture and flavor, which is vital for quality. Biosensors monitor lactic acid production in real-time, verifying cooperation [15]. Such tools optimize fermentation, maintaining ecological balance and product standards.

### 4.3. Quorum Sensing (QS)

QS, a density-dependent communication system, regulates microbial behaviors impacting food safety and shelf life, such as Pseudomonas-driven spoilage in meat or *Listeria* biofilm formation [1]. SPR biosensors detect QS signalling molecules, and while QCM sensors track the biofilm growth, providing early warnings of spoilage or contamination risks [16,28]. These insights illuminate QS’s ecological role, enabling proactive quality management. These microbial interactions—competition, cooperation, and quorum sensing—are monitored effectively by biosensors, as summarized in Table 2, which outlines key interactions (competition, cooperation, and quorum sensing), corresponding food examples, biosensor types, and detected signals. Electrochemical sensors track bacteriocins in cheese, optical (SPR) sensors detect volatile compounds in yogurt, and mechanical (QCM) sensors measure biofilm mass in meat, illustrating the role of biosensors in ecological management.

### 4.4. Fermented Food Examples

Fermented foods showcase microbial interactions at work, with biosensors ensuring their success. In fermented sausages, *Lactobacillus* spp. and *Staphylococcus* spp. synergistically contribute to preservation and flavor development, a process effectively monitored by volatile-sensing biosensors [22]. In kefir, adenosine triphosphate (ATP)-based sensors maintain yeast-*Lactobacillus* spp. balance [21]. These cases show how biosensors translate ecological interplay into reliable quality control.

## 5. Case Studies and Regional Focus

Biosensors can play a significant role in solving real-world challenges in high-risk food categories. This section presents a case study on the effective use of biosensors to address microbial issues with speed and sensitivity in the dairy, meat, and produce industries. Piezoelectric immunosensors detected *Campylobacter jejuni in dairy* at 10^2^ CFU mL^−1^ [33], graphene-based sensors identified amines in meat [19], and SPR immunosensors spotted *Salmonella* spp. at 10^3^ CFU g^−1^ in powdered milk [16]. These examples demonstrate the transformative potential of biosensors, offering actionable solutions to microbial threats.

### 5.1. Global and Regional Perspectives

Building on these cases, biosensors enable rapid and sensitive detection across dairy, meat, and produce, addressing universal safety challenges. However, their success depends on adapting to global and regional variations—climate, production practices, and AMR differ starkly across Asia, Africa, and Latin America, requiring tailored strategies. The following examples illustrate these nuances, showing how biosensors enhance food safety worldwide.

#### 5.1.1. Case Studies from Asia

Asia’s environmental and production conditions amplify microbial risks, where biosensors offer critical solutions. High temperatures in Myanmar accelerate the spoilage of meat and dairy products, leading to increased food waste and insecurity [34]. In Cambodia, AMR in livestock has surged, with a 2024 study revealing high *E. coli* resistance to ampicillin and tetracycline [35,36]. Biosensors detecting spoilage volatiles or resistant strains could transform monitoring, reduce waste, and enhance safety.

#### 5.1.2. Case Studies from Africa

In Ethiopia, 6% *Salmonella* spp. prevalence in raw milk highlights contamination risks [26]. Rapid detection using biosensors, such as SPR techniques, offers a promising solution, enabling locally tailored responses and strengthening regional food safety. In Nigeria and Kenya, microbial contamination of meat and vegetables in informal markets poses ongoing risks, with *Salmonella* and *E. coli* frequently reported [37]. Biosensors could serve as rapid tools to reduce diagnostic delays [38].

#### 5.1.3. Case Studies from Latin America

In Bolivia, governance weaknesses in AMR management necessitate the use of biosensors for resistance tracking [35], specifically for *Pseudomonas* spp. spoilage in produce and meat necessitates flexible sensors [32].

## 6. Detection and Monitoring Techniques

From a public health standpoint, biosensor-based detection and monitoring techniques have revolutionized the approach to microbial threats in food systems, outperforming traditional culturing methods and enabling rapid identification, thereby protecting populations from foodborne pathogens. Optical biosensors utilizing surface plasmon resonance were employed to detect Salmonella swiftly via antigen–antibody binding [16]. Electrochemical aptasensors have been developed for targeted detection of *Vibrio* spp. [27], and *Salmonella typhimurium* using label-free impedimetric methods [31]. Mechanical QCM sensors detect *Staphylococcus* spp. by monitoring mass changes on sensor surfaces [28]. Rapid detection of *E. coli* O157:H7 in 20 min has been achieved using a microelectrode array [7], while isothermal microcalorimetry (IMC) enables identification of *Lactobacillus plantarum* in 4.7–18.6 h across various cultivation methods [9]. Nucleic acid- and antibody-based biosensors targeting *Salmonella* spp. achieve sensitivities with limits of detection (LOD) ranging from 1 to 10^3^ CFU mL^−1^ [8]. These technologies offer high-speed results and portability, reducing the risk of foodborne outbreaks [39]. However, challenges such as matrix interference, elevated manufacturing costs, and lack of standardized protocols limit their widespread adoption [8,11], which highlights the need for refined protocols. These diverse biosensor technologies enable rapid detection across food systems, as summarized in Table 3, which summarizes detection methods, example pathogens, limits of detection (LOD), detection times, and advantages for optical (SPR), electrochemical, QCM, and bioluminescence biosensors. LODs range from 1 to 10^3^ CFU/mL, with detection times as low as 10 min, showcasing rapid, sensitive monitoring across food systems.

### 6.1. Types of Biosensors by Transduction Mechanism

Biosensors can be classified based on their transduction mechanism. Depending on the applicability, mode of operation, and targeted analyte, they are specifically selected and explored for application potential in food microbiology. Each method offers unique advantages but also has certain limitations, depending on the target analyte, food matrix, and detection environment. A few biosensors are discussed below.

#### 6.1.1. Electrochemical Biosensors

**Figure 1:**
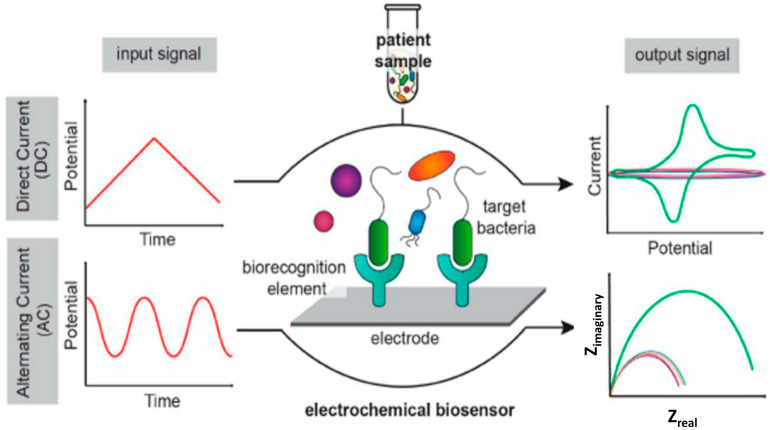
Bacterial detection using direct and alternating current-based electrochemical biosensors. Reproduced with permission from Ref. [50]. Copyright © 2020, American Chemical Society.

Electrochemical biosensors detect analytes by translating biochemical interactions into measurable electrical signals such as current, voltage, or impedance. They are widely used in food safety due to their simplicity, low cost, and compatibility with portable formats. Recent innovations, such as clustered regularly interspaced short palindromic repeats (CRISPR)-based electrochemical aptasensors, have significantly enhanced sensitivity. A schematic of bacterial detection using direct and alternating current-based electrochemical biosensors is shown in Figure 1. Cui et al. developed a CRISPR-associated protein 12a (Cas12a)– powered biosensor capable of detecting *E. coli* at the single-cell level in food samples [46]. These platforms are increasingly used to monitor pathogens, mycotoxins, and antibiotic residues [47,48,49].

#### 6.1.2. Optical Biosensors

**Figure 2:**
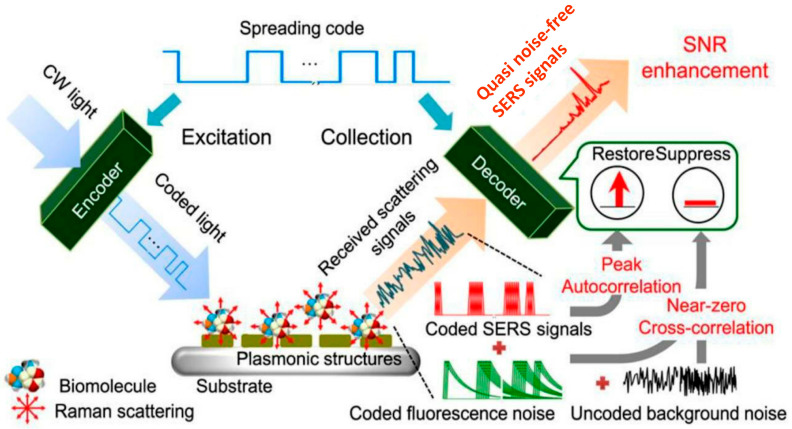
Schematic of SERS-based sensing mechanism. This figure highlights how signal enhancement occurs on nanostructured surfaces for the sensitive detection of foodborne pathogens. Reproduced with permission from Ref. [54]. Copyright © 2024 Elsevier Ltd.

Optical biosensors operate by detecting changes in light properties—such as fluorescence, absorbance, or refractive index—upon the recognition of a target. Techniques like SPR provide real-time, label-free detection of pathogens, including Salmonella [51,52], while surface-enhanced Raman scattering (SERS) (Figure 2) enables multiplex and ultra-sensitive detection in complex matrices [53].

#### 6.1.3. Piezoelectric Biosensors

**Figure 3:**
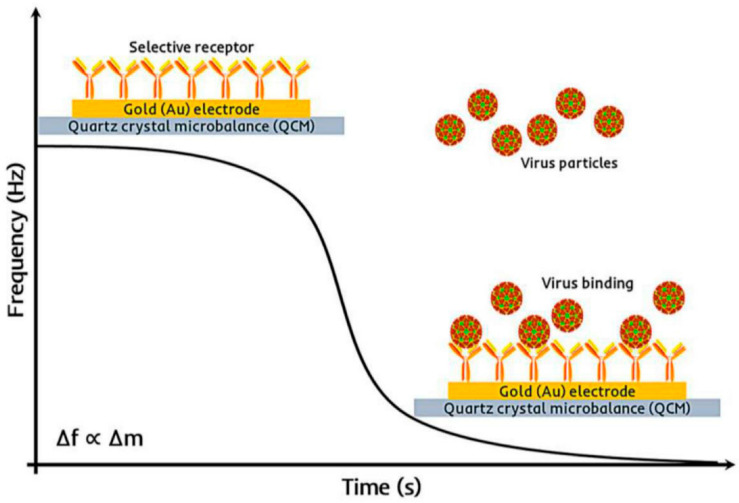
The principle of a QCM-based gravimetric virus sensor. The change in mass in response to virus binding with the selective receptor is detected as a change in the frequency of the QCM transducer. Reproduced with permission from Ref. [57]. Copyright © 2024 Elsevier Ltd.

Piezoelectric biosensors, including quartz QCM devices (Figure 3) and surface acoustic wave (SAW) devices, measure the mass change that occurs when a target binds to a sensor-coated surface. These systems provide label-free, real-time monitoring and have been successfully applied to detect bacteria such as Staphylococcus and Campylobacter in meat and dairy products [55,56]. Advances in SAW technology have improved robustness and integration with microfluidic systems.

#### 6.1.4. Thermal Biosensors

**Figure 4:**
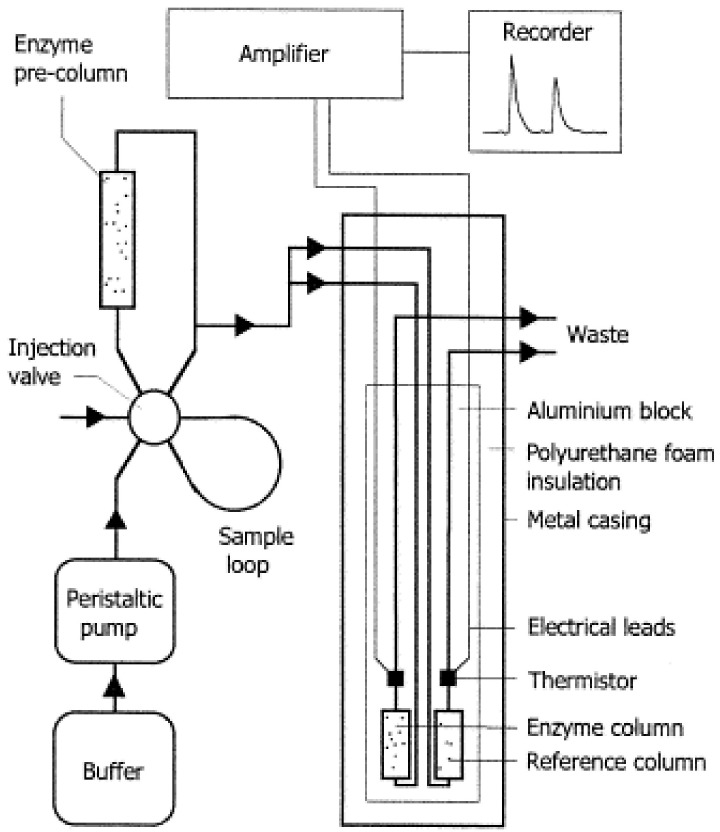
The standard structure of calorimetric biosensors. Reproduced with permission from Ref. [60]. Copyright © 2001 Elsevier Ltd.

Thermal biosensors detect temperature changes resulting from biochemical reactions or microbial metabolic activity, as shown in Figure 4. Enzyme-based thermistors have been employed to measure spoilage indicators or pesticide residues in food products. Moreover, calorimetric chips now offer sensitive, real-time detection of microbial contamination by monitoring the heat produced during bacterial growth [58,59].

#### 6.1.5. Field-Effect Transistor (FET)

**Figure 5:**
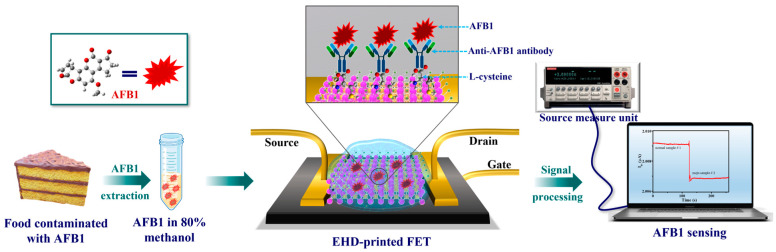
Field effect transistor-based sensor working for the detection of aflatoxin B1 from contaminated food. Reproduced with permission from Ref. [43]. Copyright © 2024 Elsevier Ltd.

FET-based biosensors utilize semiconductor interfaces to detect changes in surface potential resulting from the binding of charged analytes, as illustrated in Figure 5. Their miniaturization potential and exceptional sensitivity make them particularly suitable for real-time, label-free detection [59]. Recent developments in graphene- and MoS_2_-based FETs have enabled the rapid detection of *E. coli*, *Salmonella*, aflatoxins, and antibiotic residues with minimal sample preparation [43,61,62,63].

#### 6.1.6. Lateral Flow Assay (LFA)

**Figure 6:**
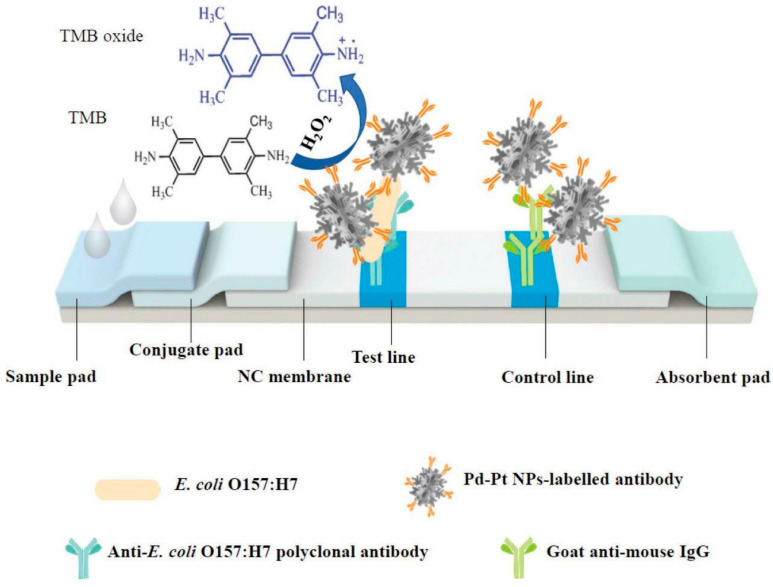
Schematic illustration of the LFA for the detection of *Escherichia coli* O157:H7. TMB = 3,3′,5,5′-tetramethylbenzidine; NC = nitrocellulose; NP = nanoparticles. Reproduced with permission from Ref. [66]. Copyright © 2024 Elsevier Ltd.

LFA-based biosensors represent one of the most accessible and widely used platforms for field-based testing. A schematic illustration of the LFA for pathogen detection is shown in Figure 6. Typically paper-based and capillary-driven, LFAs are valued for their speed, low cost, and user-friendliness. Contemporary LFAs incorporate aptamers, quantum dots, or SERS nanotags to enhance sensitivity and allow multiplexing [64]. For example, an aptamer-based LFA for aflatoxin B_1_ in cereals achieved a detection limit below 0.1 ng.mL^−1^ [65].

Different types of biosensors offer distinct advantages and are well-suited to various food safety applications. Electrochemical biosensors are among the most cost-effective and portable, often preferred for on-site detection of pathogens. Optical sensors, especially those utilizing SPR or SERS, offer label-free, real-time monitoring with high specificity; however, they typically require more complex instrumentation. Piezoelectric sensors, such as QCM, are highly sensitive and label-free, but they may be susceptible to environmental noise. FET-based biosensors stand out for their ultra-sensitivity and potential for miniaturization, making them suitable for real-time electronic integration. Thermal biosensors and calorimetric devices offer real-time metabolic monitoring but are slower in detection compared to other platforms. Lateral flow assays (LFAs), though less sensitive than lab-based methods, are widely adopted for field use due to their simplicity, speed, and cost-effectiveness. The selection of biosensor type depends on the target analyte, the required detection time, the sample matrix complexity, and resource availability.

In summary, these biosensor technologies offer tailored solutions to meet the diverse detection needs of microbial ecology in food systems. Their strategic deployment enhances early warning capabilities, improves food quality control, and supports safer supply chains.

## 7. Control Strategies Based on Microbial Ecology

Microbial ecology-based control strategies enhance food safety by using natural microbial interactions. For instance, ATP-based biosensors effectively monitored *Lactobacillus* spp. during yogurt fermentation, supporting biopreservation and reducing contamination risks [67]. Flexible biosensors detected volatile amines to mitigate meat spoilage [19], while predictive models integrated with biosensor data maintained milk quality by anticipating microbial shifts [68]. IoT-connected biosensors forecast microbial dynamics in sausage production, enhancing safety [10]. Bacteriocins suppressed pathogens, probiotics outcompeted spoilage organisms, and biosensors facilitated the rapid detection of *Salmonella* spp. detection during contamination events [8]. However, challenges such as matrix interference and standardization gaps reduce reliability [5], and high costs limit accessibility in resource-constrained regions, indicating that broader adoption depends on addressing these barriers.

### 7.1. Bacteriocins

Bacteriocins, ribosomally synthesized antimicrobial peptides produced by *Lactobacillus* spp. and other microbes, enhance biopreservation by complementing biosensor-driven strategies [69]. Peptides such as nisin and pediocin inhibit pathogens like *Listeria* spp. without affecting beneficial microflora, earning Generally Recognized as Safe (GRAS) status [70]. Widely applied in dairy, meat, and vegetable fermentations, they strengthen traditional sanitation efforts. Recent studies have revealed their synergy with biosensors, enabling real-time pathogen monitoring and guided targeted bacteriocin use, thereby reducing additive reliance while ensuring safety [69]. For instance, a high-throughput method using live fluorescent biosensors to identify potential bacteriocin producers from an extensive strain library has been developed, facilitating the selection of effective strains for food preservation [71]. Similarly, electrochemical biosensors are used to detect nisin activity in dairy products, ensuring effective pathogen suppression [72]. Furthermore, electrochemical biosensors have been used to detect lactate produced by lactic acid bacteria, which are often bacteriocin producers, providing an indirect measure of their activity in food matrices [73]. These advancements highlight the potential of biosensors in monitoring and optimizing bacteriocin use for enhanced food safety, aligning ecological principles with practical quality preservation, and minimizing chemical dependence.

### 7.2. Biofilms

Biofilms, complex microbial communities embedded in extracellular matrices, pose significant challenges by enhancing the resilience of pathogens [13]. Pathogens such as *Listeria monocytogenes* and *Salmonella* spp. form biofilms on food contact surfaces, resisting sanitizers and posing a threat to safety [74]. Biosensors detect early biofilm formation through electrochemical or optical signals, enabling timely interventions to prevent the development of mature biofilms [13]. Bacteriocin treatments, combined with physical cleaning, disrupt nascent biofilms [69]. Electrochemical biosensors integrated on microfluidic chips offer high sensitivity and real-time monitoring of microbial biofilms in food processing environments, which is crucial for preventing pathogen persistence [75]. This dual approach—real-time feedback and natural antimicrobial action—anchors biofilm management in microbial ecology, offering proactive safety solutions.

### 7.3. Probiotics

Probiotics utilize microbial ecology to enhance food safety and quality, offering sustainable benefits that extend beyond fermentation [76]. Defined as live microorganisms conferring health benefits when administered in adequate amounts [77], probiotics include *Lactobacillus* spp., *Bifidobacterium* spp., certain *Bacillus* spp., *Enterococcus* spp., and yeast species [78]. Probiotics inhibit pathogens through competitive exclusion, produce metabolites to strengthen antimicrobial barriers, and modulate host immunity [76]. Co-fermentation with probiotics shifts microbial communities away from spoilage organisms, extending shelf life [12]. Biosensors can monitor probiotic viability in real-time, ensuring optimal fermentation [67]. Certain probiotic strains also counter biofilms by disrupting adhesion, reducing sanitation costs [78].

Recent studies have demonstrated the use of bacterial biosensors, such as LUX-based and RecA-lux constructs, to evaluate probiotic strains for their antioxidant, DNA-protective, and SOS-suppressing functions, which are particularly relevant under stress or antibiotic challenge [79,80]. For example, *Lactobacillus* strains tested under intestinal-like conditions using LUX biosensors retained their protective activity, supporting their suitability for functional food applications.

Additionally, engineered probiotics equipped with synthetic biosensors are being developed to sense gastrointestinal markers (e.g., thiosulfate, heme) and trigger controlled therapeutic responses [81]. These “smart microbes” integrate diagnostic and intervention roles, offering new strategies for monitoring food and gut health. Furthermore, biosensor platforms have the potential to detect or modulate biofilm formation through probiotic-derived bacteriocins and adhesion interference, enhancing sanitation and safety [82].

This multifaceted strategy integrates probiotics and biosensors, driving safer, higher-quality products.

## 8. Decontamination Methods

Decontamination methods complement biosensor-driven detection and control, forming a robust framework for food safety grounded in microbial ecology. Thermal, non-thermal, chemical, and biocontrol techniques each address microbial risks, while biosensors pinpoint these risks, balancing safety, quality, and consumer demand for minimally processed foods. This section explores these approaches, highlighting how real-time monitoring enhances the efficacy of decontamination methods and aligns them with ecological principles.

### 8.1. Thermal Methods

Thermal processing, encompassing pasteurization and sterilization, stands as a cornerstone of microbial inactivation in food preservation [21]. These methods efficiently eliminate pathogens and spoilage organisms, but they compromise nutritional and sensory qualities—vitamin loss, color shifts, and the presence of undesirable compounds often reduce consumer appeal. Biosensors enhance thermal approaches by precisely identifying contamination risks, ensuring targeted and efficient application, thus mitigating quality trade-offs.

### 8.2. Non-Thermal Methods

Non-thermal technologies like high-pressure processing (HPP) and pulsed electric fields (PEF) overcome thermal drawbacks, delivering microbial control with minimal quality loss. HPP applies pressures of 100–600 MPa at ambient or chilled temperatures, inactivating microbes by damaging their cell membranes and proteins [83,84]. The application of HPP at 250 MPa reduces *E. coli* in orange juice without affecting sensory properties [85], while 500 MPa for 60 s effectively controls *Salmonella* in chicken meat [86]. PEF uses short, high-voltage pulses to disrupt membranes via electroporation, avoiding significant heat [87]. The efficacy of PEF against *S. senftenberg* has been demonstrated [88,89]. Biosensors optimize these methods by detecting contamination levels, ensuring precision in safety and quality management.

## 9. Chemical and Biocontrol Agents

Chemical and biocontrol agents add vital layers of protection in microbial management, enhancing physical methods and biosensor insights. Organic acid washes, including lactic, acetic, and citric acids, effectively reduce contamination in meat and produce [74]. Meanwhile, chlorine-based disinfectants, although effective, have sparked concerns over harmful by-products, prompting the development of safer alternatives. Biocontrol strategies, such as bacteriophages and antagonistic microbes, target pathogens in an eco-friendly manner, with CRISPR-based antimicrobials promising a future of precision [90]. Integrated with biosensors, these agents form a holistic approach that eliminates threats identified in real-time, preserving quality and meeting demands for sustainable, safe food.

## 10. Emerging Technologies in Food Microbiology

Emerging technologies—such as CRISPR-based assays, artificial intelligence (AI) combined with the Internet of Things (IoT), and nanotechnology-enhanced biosensors—propel food microbiology into a proactive field, building on the principles of chemical and biological control. These innovations enhance biosensor accuracy, enable predictive control, and align with this review’s focus on rapid and sensitive solutions, transforming microbial risk management.

### 10.1. CRISPR-Based Assays

CRISPR-based assays provide high-precision detection and microbial engineering. CRISPR/Cas biosensors achieved rapid detection of *Salmonella* spp. DNA/RNA in food samples within one hour, surpassing traditional methods [91]. Additionally, CRISPR-mediated modification of *Lactobacillus* spp. enhanced fermentation safety and quality [92]. This dual role positions CRISPR as a game-changer for preservation.

### 10.2. AI and IoT Systems

AI-integrated IoT systems enable predictive monitoring of microbial growth. These systems forecast microbial dynamics in meat and dairy using biosensor data [93], while AI-driven pattern recognition identifies *Listeria* spp. outbreaks [94]. Paired with biosensors, they shift strategies from reactive to proactive.

### 10.3. Blockchain for Food Traceability

Blockchain technology is being increasingly explored as a complementary tool to biosensor systems for enhancing transparency and traceability in food safety monitoring. By storing biosensor-generated data on a secure, decentralized ledger, blockchain can help ensure that microbial testing results are tamper-proof, time-stamped, and accessible across the entire supply chain. This technology not only enables faster and more informed decisions during contamination events but also supports long-term improvements in accountability and consumer trust. When used in conjunction with AI and IoT-based monitoring platforms, blockchain provides a reliable means of tracking food quality in real-time and enhancing food safety protocols from production to distribution [95].

### 10.4. Nanotechnology-Enhanced Biosensors

**Figure 7:**
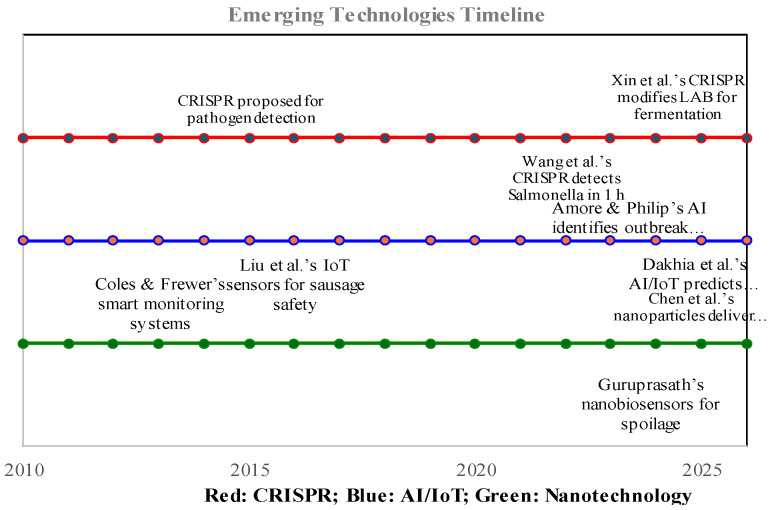
Evolution of emerging technologies in food microbiology [10,91,92,93,94,96,97,100].

Nanotechnology improves biosensor sensitivity and adaptability. Nanobiosensors detected *Pseudomonas* spp.-derived amines in meat within minutes [96], and polymer nanoparticles delivered CRISPR-based antimicrobials with high precision [97,98,99]. Continuous monitoring via innovative systems further supports real-time safety control [100]. Together, these technologies tackle AMR and regional spoilage, setting the stage for future innovations. These emerging technologies build on biosensor frameworks, with their development timeline illustrated in Figure 7, highlighting key advances in precision and predictive control. Timeline traces the development of CRISPR-based assays, AI with IoT, and nanotechnology-enhanced biosensors. Milestones include rapid Salmonella detection within an hour and predictive monitoring in meat and dairy, driving precision and proactivity in safety control.

## 11. Challenges and Future Directions

Biosensors offer immense potential in food microbiology; however, challenges temper their adoption. Complex food matrices interfere with signals [11], high costs limit access for small producers [39], and specificity struggles amid diverse microbes risk false positives [5]. Future advancements promise solutions—smart IoT/AI systems preempt risks [10], and wearable biosensors for food handlers prevent contamination, inspired by Zelada-Guillén et al. (2012) [101]. Addressing AMR remains critical, as evidenced by the detection of chloramphenicol residues using photoelectrochemical aptasensors [102] and the efficient identification of multiple pathogens via multiplexed biosensors [27]. These developments position biosensors to overcome limits and anticipate microbial challenges.

### AMR: An Expanding Frontier

AMR poses a growing, underexplored threat in food microbiology, jeopardizing safety worldwide. *Salmonella* spp. and *Campylobacter* spp. exhibit increasing resistance to antibiotics, with resistance to fluoroquinolones and cephalosporins in *S. enterica* documented in poultry and produce, and multidrug-resistant *C. jejuni* reported in European poultry [103]. The WHO’s Global Antimicrobial Resistance and Use Surveillance (GLASS) report (2025) urged enhanced surveillance, advocating biosensor tracking of resistance patterns [104]. Future biosensors must prioritize detecting AMR determinants, enabling early interventions to protect food systems and align with proactive safety goals.

## 12. Conclusions

Biosensors are transforming food microbiology by delivering rapid, real-time ecological insights across dairy, meat, and produce, redefining safety and quality control. Integrated into intelligent systems, they offer a proactive edge over traditional methods, leveraging a deeper understanding of microbes for greater impact. As detailed in this review, diverse biosensor types—ranging from electrochemical and optical to piezoelectric, thermal, FET-based, and LFA—offer tailored advantages for different food matrices and microbial targets. Challenges such as cost and matrix interference persist, but nanomaterials, IoT, and multiplexing steadily address them. Wearable biosensors and AMR detection herald a sustainable future, potentially making biosensors indispensable within a decade. This evolution bridges microbial science with global safety needs, fulfilling this review’s vision.

## Figures and Tables

**Table 1:** Predominant microbial types, roles, and biosensor applications in major food categories.

Food Category	Predominant Microbial Types	Key Roles	Biosensor Detection Examples	References
Dairy	LAB (*Lactobacillus, Lactococcus*), yeasts (*Saccharomyces*), molds (*Penicillium*)	Fermentation, flavor development, preservation	pH sensors (LAB), SPR (*Listeria*)	[2,28]
Meat/Poultry	LAB, CNS (*Staphylococcus*), spoilage bacteria (*Pseudomonas, Brochothrix*), pathogens (*Listeria, Salmonella*)	Fermentation, spoilage, safety risks	Amine sensors (*Pseudomonas*)	[7,18]
Fresh Produce	Gram-negative bacteria (*Pseudomonas, Enterobacteriaceae*), fungi, pathogens (*Salmonella*, *E. coli* O157:H7)	Spoilage, safety risks, plant adaptation	SPR (*Salmonella*)	[19,21,22]

Note: LAB: lactic acid bacteria; CNS (coagulase negative Staphylococci).

**Table 2:** Microbial interactions in food systems.

Interaction	Food Example	Biosensor Type	Detected Signal	References
Competition	Cheese	Electrochemical	Bacteriocins	[2,27]
Cooperation	Yogurt	Optical	Volatile compounds	[9,31]
Quorum sensing	Meat	QCM	Biofilm mass	[21,32]

Note: QCM: Quartz Crystal Microbalance.

**Table 3:** Biosensor detection capabilities in food microbiology.

Biosensor Type	Detection Method	Example Pathogen	LOD (CFU/mL)	Detection Time	Advantages	References
Optical (SPR)	Surface plasmon resonance	*Salmonella*	12	20 min	High sensitivity, label-free	[40]
Electrochemical	Amperometric/impedimetric	*E. coli*	0.35	30 min	Cost-effective, rapid	[41]
QCM	Mass change detection	*E. coli*	10^2^	30 min	High sensitivity, label-free	[42]
FET-based	Charge-sensitive transistor readout	Aflatoxin B1,	5.6 ppb	~2 min	Ultra-sensitive, label-free, scalable electronics	[43]
LFA (strip-based)	Lateral capillary flow, colorimetric/SERS	Aflatoxin B1, *Listeria*, *Salmonella*	<1 ng/mL; 10^3^ CFU/mL	10–20 min	Rapid, low-cost, field-deployable	[44,45]

## Data Availability

All the data are shared in the manuscript.

## References

[1] Smid E.J., Lacroix C. (2013). Microbe–microbe interactions in mixed culture food fermentations. Curr. Opin. Biotechnol..

[2] Bourdichon F., Casaregola S., Farrokh C., Frisvad J.C., Gerds M.L., Hammes W.P., Harnett J., Huys G., Laulund S., Ouwehand A. (2012). Food fermentations: Microorganisms with technological beneficial use. Int. J. Food Microbiol..

[3] Jay J.M., Loessner M.J., Golden D.A. (2008). Modern Food Microbiology.

[4] Doyle M.P., Diez-Gonzalez F., Hill C. (2020). Food Microbiology: Fundamentals and Frontiers.

[5] Law J.W.-F., Ab Mutalib N.-S., Chan K.-G., Lee L.-H. (2015). Rapid methods for the detection of foodborne bacterial pathogens: Principles, applications, advantages and limitations. Front. Microbiol..

[6] Ercolini D. (2013). High-throughput sequencing and metagenomics: Moving forward in the culture-independent analysis of food microbial ecology. Appl. Environ. Microbiol..

[7] Radke S.M., Alocilja E.C. (2005). A high density microelectrode array biosensor for detection of *E. coli* O157: H7. Biosens. Bioelectron..

[8] Lee K.-M., Runyon M., Herrman T.J., Phillips R., Hsieh J. (2015). Review of Salmonella detection and identification methods: Aspects of rapid emergency response and food safety. Food Control.

[9] Fricke C., Harms H., Maskow T. (2019). Rapid calorimetric detection of bacterial contamination: Influence of the cultivation technique. Front. Microbiol..

[10] Liu H., Zhou X., Liu W., Yang X., Xing D. (2016). based bipolar electrode electrochemiluminescence switch for label-free and sensitive genetic detection of pathogenic bacteria. Anal. Chem..

[11] Havelaar A.H., Brul S., De Jong A., De Jonge R., Zwietering M.H., Ter Kuile B.H. (2010). Future challenges to microbial food safety. Int. J. Food Microbiol..

[12] Papadopoulou O.S., Doulgeraki A., Panagou E., Argyri A.A. (2023). Recent advances and future perspective in probiotics isolated from fermented foods: From quality assessment to novel products. Front. Microbiol..

[13] Liu X., Yao H., Zhao X., Ge C. (2023). Biofilm formation and control of foodborne pathogenic bacteria. Molecules.

[14] Spacova I., Binda S., Ter Haar J.A., Henoud S., Legrain-Raspaud S., Dekker J., Espadaler-Mazo J., Langella P., Martín R., Pane M. (2023). Comparing technology and regulatory landscape of probiotics as food, dietary supplements and live biotherapeutics. Front. Microbiol..

[15] Lomakina G.Y., Modestova Y.A., Ugarova N. (2015). Bioluminescence assay for cell viability. Biochemistry.

[16] Farka Z.k., Juřík T.s., Pastucha M.j., Skládal P. (2016). Enzymatic precipitation enhanced surface plasmon resonance immunosensor for the detection of Salmonella in powdered milk. Anal. Chem..

[17] Oliver J.D. (2010). Recent findings on the viable but nonculturable state in pathogenic bacteria. FEMS Microbiol. Rev..

[18] Kračmarová M., Stiborova H., Horáčková Š., Demnerová K. (2018). Rapid Detection of Microbial Contamination in UHT Milk: Practical Application in Dairy Industry. Czech J. Food Sci..

[19] Biswas S.K., Islam M.S., Jia F., Cao Y., Li Y., Cao C. (2024). Flexible biosensors for food pathogen detection. Adv. Electron. Mater..

[20] Esmael A., Al-Hindi R.R., Albiheyri R.S., Alharbi M.G., Filimban A.A., Alseghayer M.S., Almaneea A.M., Alhadlaq M.A., Ayubu J., Teklemariam A.D. (2023). Fresh produce as a potential vector and reservoir for human bacterial pathogens: Revealing the ambiguity of interaction and transmission. Microorganisms.

[21] Zhang Y., Dong L., Zhang J., Shi J., Wang Y., Wang S. (2021). Adverse effects of thermal food processing on the structural, nutritional, and biological properties of proteins. Annu. Rev. Food Sci. Technol..

[22] Magan N., Pavlou A., Chrysanthakis I. (2001). Milk-sense: A volatile sensing system recognizes spoilage bacteria and yeasts in milk. Sens. Actuators B Chem..

[23] Mandal P.K., Biswas A.K. (2020). Modern techniques for rapid detection of meatborne pathogens. Meat Quality Analysis.

[24] De Filippis F., Parente E., Ercolini D. (2017). Metagenomics insights into food fermentations. Microb. Biotechnol..

[25] Bagheryan Z., Raoof J.-B., Golabi M., Turner A.P., Beni V. (2016). Diazonium-based impedimetric aptasensor for the rapid label-free detection of Salmonella typhimurium in food sample. Biosens. Bioelectron..

[26] Keba A., Rolon M.L., Tamene A., Dessie K., Vipham J., Kovac J., Zewdu A. (2020). Review of the prevalence of foodborne pathogens in milk and dairy products in Ethiopia. Int. Dairy J..

[27] Sha Y., Zhang X., Li W., Wu W., Wang S., Guo Z., Zhou J., Su X. (2016). A label-free multi-functionalized graphene oxide based electrochemiluminscence immunosensor for ultrasensitive and rapid detection of Vibrio parahaemolyticus in seawater and seafood. Talanta.

[28] Pohanka M. (2020). QCM immunosensor for the determination of Staphylococcus aureus antigen. Chem. Pap..

[29] Vásquez G., Rey A., Rivera C., Iregui C., Orozco J. (2017). Amperometric biosensor based on a single antibody of dual function for rapid detection of Streptococcus agalactiae. Biosens. Bioelectron..

[30] Sieuwerts S., Molenaar D., van Hijum S.A., Beerthuyzen M., Stevens M.J., Janssen P.W., Ingham C.J., de Bok F.A., de Vos W.M., van Hylckama Vlieg J.E. (2010). Mixed-culture transcriptome analysis reveals the molecular basis of mixed-culture growth in Streptococcus thermophilus and Lactobacillus bulgaricus. Appl. Environ. Microbiol..

[31] Sheikhzadeh E., CHamsaz M., Turner A., Jager E., Beni V. (2016). Label-free impedimetric biosensor for Salmonella Typhimurium detection based on poly [pyrrole-co-3-carboxyl-pyrrole] copolymer supported aptamer. Biosens. Bioelectron..

[32] Snyder A.B., Martin N., Wiedmann M. (2024). Microbial food spoilage: Impact, causative agents and control strategies. Nat. Rev. Microbiol..

[33] Wang H., Wang L., Hu Q., Wang R., Li Y., Kidd M. (2018). Rapid and sensitive detection of Campylobacter jejuni in poultry products using a nanoparticle-based piezoelectric immunosensor integrated with magnetic immunoseparation. J. Food Prot..

[34] FAO (2023). The Impact of Disasters on Agriculture and Food Security 2023—Avoiding and Reducing Losses Through Investment in Resilience.

[35] Food and Agriculture Organization of the United Nations (2024). WAAW/ACT Project is Helping the Plurinational State of Bolivia Tackle the Threat of Foodborne AMR. https://www.fao.org/fao-who-codexalimentarius/news-and-events/news-details/en/c/1724299/.

[36] (2025). Action to Support Implementation of Codex AMR Texts (ACT) Project: Progress in Cambodia.

[37] Somorin Y.M., Odeyemi O.A., Ateba C.N. (2021). Salmonella is the most common foodborne pathogen in African food exports to the European Union: Analysis of the Rapid Alert System for Food and Feed (1999–2019). Food Control.

[38] Wang B., Wang H., Lu X., Zheng X., Yang Z. (2023). Recent advances in electrochemical biosensors for the detection of foodborne pathogens: Current perspective and challenges. Foods.

[39] Ali A.A., Altemimi A.B., Alhelfi N., Ibrahim S.A. (2020). Application of biosensors for detection of pathogenic food bacteria: A review. Biosensors.

[40] Bhandari D., Chen F.-C., Bridgman R.C. (2022). Magnetic nanoparticles enhanced surface Plasmon resonance biosensor for rapid detection of Salmonella Typhimurium in Romaine lettuce. Sensors.

[41] Fande S., Amreen K., Sriram D., Goel S. (2023). Microfluidic electrochemical device for real-time culturing and interference-free detection of *Escherichia coli*. Anal. Chim. Acta.

[42] Forinová M., Pilipenco A., Lynn N.S., Obořilová R., Šimečková H., Vrabcová M., Spasovová M., Jack R., Horák P., Houska M. (2024). A reusable QCM biosensor with stable antifouling nano-coating for on-site reagent-free rapid detection of *E. coli* O157: H7 in food products. Food Control.

[43] Siva S., Bodkhe G.A., Cong C., Kim S.H., Kim M. (2024). Electrohydrodynamic-printed ultrathin Ti3C2Tx-MXene field-effect transistor for probing aflatoxin B1. Chem. Eng. J..

[44] Xing G., Sun X., Li N., Li X., Wu T., Wang F. (2022). New advances in lateral flow immunoassay (LFI) technology for food safety detection. Molecules.

[45] Kakkar S., Gupta P., Yadav S.P.S., Raj D., Singh G., Chauhan S., Mishra M.K., Martín-Ortega E., Chiussi S., Kant K. (2024). Lateral flow assays: Progress and evolution of recent trends in point-of-care applications. Mater. Today Bio.

[46] Cui J., Luo Q., Wei C., Deng X., Liang H., Wei J., Gong Y., Tang Q., Zhang K., Liao X. (2024). Electrochemical biosensing for *E. coli* detection based on triple helix DNA inhibition of CRISPR/Cas12a cleavage activity. Anal. Chim. Acta.

[47] Frigoli M., Krupa M.P., Hooyberghs G., Lowdon J.W., Cleij T.J., Diliën H., Eersels K., van Grinsven B. (2024). Electrochemical Sensors for Antibiotic Detection: A Focused Review with a Brief Overview of Commercial Technologies. Sensors.

[48] Flores-Ramírez A.Y., González-Estrada R.R., Chacón-López M.A., de Lourdes García-Magaña M., Montalvo-González E., Álvarez-López A., Rodríguez-López A., López-García U.M. (2024). Detection of foodborne pathogens in contaminated food using nanomaterial-based electrochemical biosensors. Anal. Biochem..

[49] Szelenberger R., Cichoń N., Zajaczkowski W., Bijak M. (2024). Application of Biosensors for the Detection of Mycotoxins for the Improvement of Food Safety. Toxins.

[50] Karbelkar A.A., Furst A.L. (2020). Electrochemical diagnostics for bacterial infectious diseases. ACS Infect. Dis..

[51] Yashini M., Shanmugasundaram S., Sunil C. (2024). Surface plasmon biosensing for the detection of foodborne pathogens. Biosensors for Foodborne Pathogens Detection.

[52] Yang Q., Zu J., Zhang S., Liu C., Qin X., Xu W. (2024). An overview of rapid detection methods for Salmonella. Food Control.

[53] Rahman M.H.-U., Sikder R., Tripathi M., Zahan M., Ye T., Gnimpieba Z. E., Jasthi B.K., Dalton A.B., Gadhamshetty V. (2024). Machine learning-assisted raman spectroscopy and SERS for bacterial pathogen detection: Clinical, food safety, and environmental applications. Chemosensors.

[54] Tran V.A., Tran T.T.V., Doan V.D., Vo G.N., Tran V.H., Jeong H., Vo T.T.T. (2024). Advanced nano engineering of surface-enhanced Raman scattering technologies for sensing applications. Appl. Mater. Today.

[55] Länge K. (2022). Bulk and surface acoustic wave biosensors for milk analysis. Biosensors.

[56] Zeng Y., Yuan R., Fu H., Xu Z., Wei S. (2024). Foodborne pathogen detection using surface acoustic wave biosensors: A review. RSC Adv..

[57] Afzal A., Mujahid A., Schirhagl R., Bajwa S.Z., Latif U., Feroz S. (2017). Gravimetric viral diagnostics: QCM based biosensors for early detection of viruses. Chemosensors.

[58] Papkovsky D.B., Kerry J.P. (2023). Oxygen sensor-based respirometry and the landscape of microbial testing methods as applicable to food and beverage matrices. Sensors.

[59] Das D. (2021). Measuring Metabolism and Bioenergetic Profiles of Biofilm: Isothermal Calorimetry, Differential Scanning Calorimetry, and Future of Chip Calorimetry. Anal. Methodol. Biofilm Res..

[60] Ramanathan K., Danielsson B. (2001). Principles and applications of thermal biosensors. Biosens. Bioelectron..

[61] Alvandi H., Asadi F., Rezayan A.H., Hajghassem H., Rahimi F. (2025). Ultrasensitive biosensor based on MXene-GO field-effect transistor for the rapid detection of endotoxin and whole-cell *E. coli* in human blood serum. Anal. Chim. Acta.

[62] Foyez T., Imran A.B. (2025). Electrochemical Nanobiosensors Approaches for Rapid Diagnosis of Infectious Diseases. Nano-Biosens. Technol. Diagn. Infect. Dis..

[63] Feng X., Li P., Xiao M., Li T., Chen B., Wang X., Wang L. (2024). Recent advances in the detection of pathogenic microorganisms and toxins based on field-effect transistor biosensors. Crit. Rev. Food Sci. Nutr..

[64] Li Z., Jallow A., Nidiaye S., Huang Y., Zhang Q., Li P., Tang X. (2024). Improvement of the sensitivity of lateral flow systems for detecting mycotoxins: Up-to-date strategies and future perspectives. Compr. Rev. Food Sci. Food Saf..

[65] Zhu C., Zhang G., Huang Y., Yang S., Ren S., Gao Z., Chen A. (2018). Dual-competitive lateral flow aptasensor for detection of aflatoxin B1 in food and feedstuffs. J. Hazard. Mater..

[66] Han J., Zhang L., Hu L., Xing K., Lu X., Huang Y., Zhang J., Lai W., Chen T. (2018). Nanozyme-based lateral flow assay for the sensitive detection of *Escherichia coli* O157: H7 in milk. J. Dairy Sci..

[67] Zhang Z., Wang C., Zhang L., Meng Q., Zhang Y., Sun F., Xu Y. (2017). Fast detection of *Escherichia coli* in food using nanoprobe and ATP bioluminescence technology. Anal. Methods.

[68] Ziyaina M., Rasco B., Sablani S.S. (2020). Rapid methods of microbial detection in dairy products. Food Control.

[69] Putri D.A., Lei J., Rossiana N., Syaputri Y. (2024). Biopreservation of Food Using Bacteriocins From Lactic Acid Bacteria: Classification, Mechanisms, and Commercial Applications. Int. J. Microbiol..

[70] Pujato S.A., Mercanti D.J., Briggiler Marcó M., Capra M.L., Quiberoni A., Guglielmotti D.M. (2024). Bacteriocins from lactic acid bacteria: Strategies for the bioprotection of dairy foods. Front. Food Sci. Technol..

[71] Otto S.J., Teichmann L., Fante N., Crauwels P., Grünberger A., Neddermann T., Riedel C.U. (2024). High-throughput detection of potential bacteriocin producers in a large strain library using live fluorescent biosensors. Front. Bioeng. Biotechnol..

[72] Zhang Z., Zhou J., Du X. (2019). Electrochemical biosensors for detection of foodborne pathogens. Micromachines.

[73] Ozoglu O., Uzunoglu A., Unal M.A., Gumustas M., Ozkan S.A., Korukluoglu M., Altuntas E.G. (2023). Electrochemical detection of lactate produced by foodborne presumptive lactic acid bacteria. J. Biosci. Bioeng..

[74] Nazir A., Ochani S., Nazir A., Fatima B., Ochani K., Al Hasibuzzaman M., Ullah K. (2023). Rising trends of foodborne illnesses in the US. Ann. Med. Surg..

[75] Abouhagger A., Celiešiūtė-Germanienė R., Bakute N., Stirke A., Melo W.C. (2024). Electrochemical biosensors on microfluidic chips as promising tools to study microbial biofilms: A review. Front. Cell. Infect. Microbiol..

[76] Latif A., Shehzad A., Niazi S., Zahid A., Ashraf W., Iqbal M.W., Rehman A., Riaz T., Aadil R.M., Khan I.M. (2023). Probiotics: Mechanism of action, health benefits and their application in food industries. Front. Microbiol..

[77] Hill C., Guarner F., Reid G., Gibson G.R., Merenstein D.J., Pot B., Morelli L., Canani R.B., Flint H.J., Salminen S. (2014). Activity of cecropin P1 and FA-LL-37 against urogenital microflora. Nat. Rev. Gastroenterol. Hepatol..

[78] Sarita B., Samadhan D., Hassan M.Z., Kovaleva E.G. (2025). A comprehensive review of probiotics and human health-current prospective and applications. Front. Microbiol..

[79] Chistyakov V.A., Prazdnova E.V.e., Mazanko M.S., Bren A.B. (2018). The use of biosensors to explore the potential of probiotic strains to reduce the SOS response and mutagenesis in bacteria. Biosensors.

[80] Mazanko M., Prazdnova E., Kulikov M., Maltseva T., Rudoy D., Chikindas M. (2022). Antioxidant and antimutagenic properties of probiotic Lactobacilli determined using LUX-biosensors. Enzym. Microb. Technol..

[81] Rottinghaus A.G., Amrofell M.B., Moon T.S. (2020). Biosensing in smart engineered probiotics. Biotechnol. J..

[82] Barra M., Danino T., Garrido D. (2020). Engineered probiotics for detection and treatment of inflammatory intestinal diseases. Front. Bioeng. Biotechnol..

[83] Balasubramaniam V., Martinez-Monteagudo S.I., Gupta R. (2015). Principles and application of high pressure–based technologies in the food industry. Annu. Rev. Food Sci. Technol..

[84] Rastogi N., Raghavarao K., Balasubramaniam V., Niranjan K., Knorr D. (2007). Opportunities and challenges in high pressure processing of foods. Crit. Rev. Food Sci. Nutr..

[85] Bulut S., Karatzas K.A. (2021). Inactivation of *Escherichia coli* K12 in phosphate buffer saline and orange juice by high hydrostatic pressure processing combined with freezing. LWT.

[86] Cap M., Paredes P.F., Fernández D., Mozgovoj M., Vaudagna S.R., Rodriguez A. (2020). Effect of high hydrostatic pressure on Salmonella spp inactivation and meat-quality of frozen chicken breast. LWT.

[87] Jeyamkondan S., Jayas D., Holley R. (1999). Pulsed electric field processing of foods: A review. J. Food Prot..

[88] Álvarez I., Raso J., Palop A., Sala F.J. (2000). Influence of different factors on the inactivation of Salmonella senftenberg by pulsed electric fields. Int. J. Food Microbiol..

[89] Barba F.J., Parniakov O., Pereira S.A., Wiktor A., Grimi N., Boussetta N., Saraiva J.A., Raso J., Martin-Belloso O., Witrowa-Rajchert D. (2015). Current applications and new opportunities for the use of pulsed electric fields in food science and industry. Food Res. Int..

[90] Xie S., Yue Y., Yang F. (2024). Recent Advances in CRISPR/Cas System-Based Biosensors for the Detection of Foodborne Pathogenic Microorganisms. Micromachines.

[91] Wang H., Wu Q., Zhou M., Li C., Yan C., Huang L., Qin P. (2022). Development of a CRISPR/Cas9-integrated lateral flow strip for rapid and accurate detection of Salmonella. Food Control.

[92] Xin Y., Guo T., Qiao M. (2025). Current application and future prospects of CRISPR-Cas in lactic acid Bacteria: A review. Food Res. Int..

[93] Dakhia Z., Russo M., Merenda M. (2025). AI-Enabled IoT for Food Computing: Challenges, Opportunities, and Future Directions. Sensors.

[94] Amore A., Philip S. (2023). Artificial intelligence in food biotechnology: Trends and perspectives. Front. Ind. Microbiol..

[95] Galvez J.F., Mejuto J.C., Simal-Gandara J. (2018). Future challenges on the use of blockchain for food traceability analysis. TrAC Trends Anal. Chem..

[96] Guruprasath N., Sankarganesh P., Adeyeye S., Babu A.S., Parthasarathy V. (2024). Review on emerging applications of nanobiosensor in food safety. J. Food Sci..

[97] Chen Y., Wang Y., Zhang Y., Wang X., Zhang C., Cheng N. (2024). Intelligent biosensors promise smarter solutions in food safety 4.0. Foods.

[98] Mitchell M.J., Billingsley M.M., Haley R.M., Wechsler M.E., Peppas N.A., Langer R. (2021). Engineering precision nanoparticles for drug delivery. Nat. Rev. Drug Discov..

[99] Sun Y., Chatterjee S., Lian X., Traylor Z., Sattiraju S.R., Xiao Y., Dilliard S.A., Sung Y.-C., Kim M., Lee S.M. (2024). In vivo editing of lung stem cells for durable gene correction in mice. Science.

[100] Coles D., Frewer L.J. (2013). Nanotechnology applied to European food production–A review of ethical and regulatory issues. Trends Food Sci. Technol..

[101] Zelada-Guillén G.A., Sebastián-Avila J.L., Blondeau P., Riu J., Rius F.X. (2012). Label-free detection of Staphylococcus aureus in skin using real-time potentiometric biosensors based on carbon nanotubes and aptamers. Biosens. Bioelectron..

[102] Zhou Y., Sui C., Yin H., Wang Y., Wang M., Ai S. (2018). Tungsten disulfide (WS 2) nanosheet-based photoelectrochemical aptasensing of chloramphenicol. Microchim. Acta.

[103] European Food Safety Authority, European Centre for Disease Prevention and Control (2025). The European Union summary report on antimicrobial resistance in zoonotic and indicator bacteria from humans, animals and food in 2022–2023. EFSA J..

[104] World Health Organization (2022). Global Antimicrobial Resistance and Use Surveillance System (GLASS) Report 2022.

